# Adjusting for outcome risk factors in immigrant datasets: total or direct effects?

**DOI:** 10.1186/s12874-023-01861-4

**Published:** 2023-02-10

**Authors:** Roy Miodini Nilsen, Kari Klungsøyr, Hein Stigum

**Affiliations:** 1 Faculty of Health and Social Sciences, Western Norway University of Applied Sciences, Bergen, Norway; 2 Department of Global Public Health and Primary Care, University of Bergen, Bergen, Norway; 3 Division of Mental and Physical Health, Norwegian Institute of Public Health, Bergen, Norway; 4 Department of Community Medicine and Global Health, University of Oslo, Oslo, Norway

**Keywords:** Immigrant, Ethnicity, Confounding, Adjustment, Mediator, Total effect, Controlled direct effect, Directed acyclic graphs, Collider bias, Selection bias

## Abstract

**Background:**

When quantifying differences in health outcomes between immigrants and non-immigrants, it is common practice to adjust for observed differences in outcome risk factors between the groups being compared. However, as some of these outcome risk factors may act as mediators on the causal path between the exposure and outcome, adjusting for these may remove effects of factors that characterize the immigrants rather than removing a bias between immigrants and non-immigrants.

**Methods:**

This study investigates the underlying conditions for which adjusting for outcome risk factors in regression models can lead to the estimation of either total or direct effect for the difference in health outcomes between immigrants and non-immigrants. For this investigation, we use modern tools in causal inference to construct causal models that we believe are highly relevant in an immigrant dataset. In these models, the outcome risk factor is modeled either as a mediator, a selection factor, or a combined mediator/selection factor. Unlike mediators, selection factors are variables that affect the probability of being in the immigrant dataset and may contribute to a bias when comparing immigrants and non-immigrants.

**Results:**

When the outcome risk factor acts both as a mediator and selection factor, the adjustment for the risk factor in regression models leads to the estimation of what is known as a “controlled” direct effect. When the outcome risk factor is either a selection factor or a mediator alone, the adjustment for the risk factor in regression models leads to the estimation of a total effect or a controlled direct effect, respectively. In all regression analyses, also adjusting for various confounding paths, including mediator-outcome confounding, may be necessary to obtain valid controlled direct effects or total effects.

**Conclusions:**

Depending on the causal role of the outcome risk factors in immigrant datasets, regression adjustment for these may result in the estimation of either total effects or controlled direct effects for the difference in outcomes between immigrants and non-immigrants. Because total and controlled direct effects are interpreted differently, we advise researchers to clarify to the readers which types of effects are presented when adjusting for outcome risk factors in immigrant datasets.

## Background

In recent years, there has been a significant increase in the number of studies examining differences in health outcomes between immigrants and non-immigrants [1–10]. In most of these studies, the differences are quantified by using regression models where the individual’s country or region of birth is the exposure. In addition to the exposure, several other variables associated with the outcome, such as age, education, overweight, and smoking, are included in the regression models [4–10]. The basic idea for including these is to adjust for potential bias that may arise due to the observed differences in these outcome risk factors between the compared groups.

However, the unequal distribution of an outcome risk factor between compared groups may not always represent a bias. A difference in the outcome risk factor may also arise because immigrants and non-immigrants already predispose to a difference in the risk factor before immigration took place. For example, individuals from the migrating country may, in general, smoke cigarettes less often than the host population they move to. In this situation, the outcome risk factor becomes a mediator [11], in that the difference in the distribution of smoking between the migrant and host countries represents an association that is of interest for understanding the differences in health outcomes between immigrants and non-immigrants.

Because outcome risk factors may act as mediators on the causal path between the exposure and outcome, the adjustment for such risk factors in regression models can lead to the estimation of a direct effect rather than a total effect [11–13]. In general, the direct effect refers to the association between the exposure and outcome unexplained by mediators lying on the causal path between the exposure and outcome, whereas the total effect refers to the association between the exposure and outcome without considering such mediators [11–13]. The total effect is the type of effect authors typically want to estimate when comparing immigrants and non-immigrants in terms of health outcomes.

In this study, we use modern tools in causal inference to investigate the underlying conditions leading to unequal distributions in the outcome risk factors between immigrants and non-immigrants. We then show how the adjustment for outcome risk factors in regression models under these conditions can lead to the estimation of either total or direct effects. A summary of our results is presented in a tabulated form to guide researchers that aim to report the correct effect type in their specific studies.

## Methods

### Causal interpretation

In causal inference, it is presumed that the exposure under study can be manipulated in the same way as a treatment assignment in a randomized controlled trial [14]. For country of birth and similar variables (e.g., sex and race), however, this manipulation cannot be directly performed because such variables do not correspond to clearly defined interventions [14–17]. To solve this, some authors suggest alternative representations of these exposures by specifying relevant components that are hypothetically manipulable [16, 17]. For example, if skin cancer is our outcome and country of birth is our exposure, we could let country of birth represent the joint effect of skin color and genes. Although interventions that would change these components are generally not feasible, describing such interventions can help clarify the causal interpretation of the effect estimates when contrasting country of birth [16, 17]. In this study, we do not specify the hypothesized components of country of birth, but we assume that they can be defined.

### Directed acyclic graphs

To model the association between country of birth and a health outcome, we use directed acyclic graphs (DAG), which is a graphical tool for conducting causal inference in epidemiologic research [18]. Although DAGs have been applied in connection with health inequalities across race groups before [16, 19], there is little published information on how DAGs are applied to immigrant data.

In causal DAGs, we denote the variables nodes and let arrows between nodes represent causal effects. In brief, an exposure $E$ has a direct effect on the outcome $D$ if the two variables are connected with a single directed arrow $(E\to D)$. A variable can also have an indirect effect on another variable ($E\to M\to D$) via a mediator $M$. When a variable $C$ points at two other variables ($E\leftarrow C\to D$), the variable is defined as a common cause and corresponds to what epidemiologists call a confounding factor [20]. When two variables point at the same variable ($E\to S\leftarrow D$), the variable $S$ is defined as a common effect or “collider” [18, 21, 22].

All above paths are defined as “open” except for the collider path, which is defined as a “closed” path. Open paths represent statistical associations, whereas closed or no paths represent the absence of associations. When controlling for a mediator or a confounding factor, for example, by regression adjustment or conditioning on a single variable value, the paths that were originally open get closed. If we, on the other hand, control for a collider, we open the path that originally was closed by the collider. Importantly, both confounding paths and collider paths should be closed to avoid biased associations between variables. When opening a collider path, the induced bias is usually called collider bias or selection bias [18, 21–23].

### Immigrant datasets

**Fig. 1:**
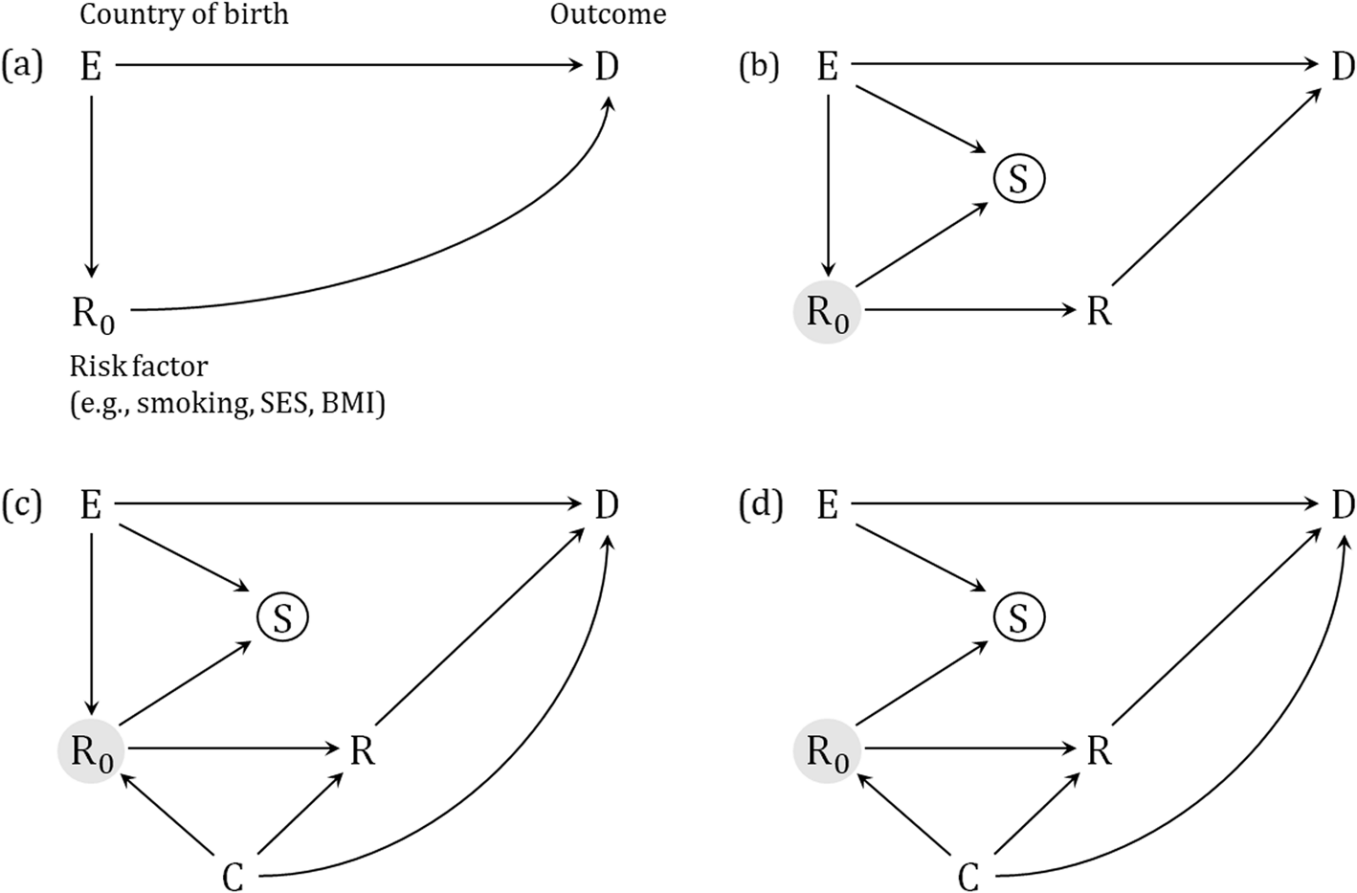
Directed acyclic graphs. The association between country of birth ($\mathrm{E}$) and a health outcome ($\mathrm{D}$) involving an outcome risk factor ($\mathrm{R}$). A gray node indicates an unmeasured variable. See details in the text for interpretations. Abbreviations: SES, socioeconomic status; BMI, body mass index

Figure 1a represents a causal DAG for the association between country of birth and a health outcome before immigration takes place. We later extend this model to also include the more complex collider paths forming the basis of an immigrant dataset. For now, we will use the initial model to introduce variables, causal directions of variables, and effect types.

In Fig. 1a, we have assumed national representative data for two or more countries $E$ (e.g., countries A and B) and that only one risk factor ${R}_{0}$ is present for the health outcome $D$. The arrows from $E$ to $D$ and ${R}_{0}$ to $D$ indicate that country of birth and the risk factor affect the outcome directly. We also suggest a national difference in the distribution of the outcome risk factor ${R}_{0}$ between the countries being compared, indicated by the arrow from $E$ to ${R}_{0}$. An example could be that individuals from one country smoke cigarettes more often than individuals from the other country. The outcome risk factor, ${R}_{0}$, could also represent socioeconomic status (SES), body mass index (BMI), body height, or nutritional status.

Note that, the direction of the arrow for the national difference in the distribution of ${R}_{0}$ between the countries being compared is going from $E$ to ${R}_{0}$ ($E\to {R}_{0}$), and not the other way around. The reason for this is that no known or unknown factor can influence which country one is born in, except perhaps factors that influence where the parents decide to live. Therefore, any observed difference in the distribution of an outcome risk factor ${R}_{0}$ between two or more countries must be inherent and specific for the compared countries, for example, due to the countries’ culture, tradition, genetic composition, or socioeconomic position. In this regard, it may be advantageous to have some knowledge of demographics in the relevant countries to be compared.

In Fig. 1a, we assumed national representative data of the countries being compared. However, datasets comparing immigrants and non-immigrants often consist of national representative data for the receiving country (non-immigrants) but subsamples of individuals for the migrating countries (immigrants). In addition, there may be a selection on the outcome risk factor ${R}_{0}$ among those who migrated. That is, the distribution of ${R}_{0}$ may differ between those who migrate and those who did not migrate from the migrating country. For example, resourceful individuals with higher SES might find it “easier” to migrate than those less resourceful with lower SES.

In Fig. 1b, we have introduced a binary variable $S$ to indicate whether an individual is a member of the dataset to be analyzed (the immigrant dataset). If we let country A be the receiving country and country B the migrating country, then the immigrant dataset ($S=1$) would contain the representative set of individuals from country A (non-immigrants) as well as a small subsample of individuals from country B (immigrants). Further, because the probability of being a member of the immigrant dataset naturally differs between country A and country B, we present an arrow from $E$ to $S$. Similarly, because the probability of being a member of the immigrant dataset may depend on the outcome risk factor ${R}_{0}$ (e.g., SES or BMI), we also present an arrow from ${R}_{0}$ to $S$. The outcome risk factor will then also be a “selection factor” for being in the immigrant dataset.

Further, in immigrant datasets, the outcome risk factor is usually measured post-migration, often in connection with a health survey in the receiving country. Therefore, in Fig. 1b, we introduce $R$ as the measured risk factor post-migration whereas ${R}_{0}$ represents the same unmeasured risk factor before migration took place. Note that, as ${R}_{0}$ is an ancestor of $R$, the measured risk factor will lay in the causal path between ${R}_{0}$ and the outcome $D$ (${R}_{0}\to R\to D$). However, we will not present an arrow from $R$ to $S$ as the risk factor is measured post-migration and can, therefore, not itself be a direct cause for being a member of the immigrant dataset $S$.

### Total effect and controlled direct effect

When comparing immigrants and non-immigrants in terms of health outcomes, the goal is often to estimate the total effects. That is, we aim to estimate the effect of $E$ on $D$ without separating the different paths that could explain the effect of country of birth on the outcome. In general, the paths of the total effect can be separated into direct and indirect effects. The indirect effect refers to the part of the total effect that is explained by a given set of mediators, whereas the direct effect refers to the part of the total effect that is unexplained by the same mediators [11–13]. For example, in Fig. 1a, the total effect of $E$ on $D$ is composed of the direct effect $E\to D$ and the indirect effect $E\to {R}_{0}\to D$, where ${R}_{0}$ is a mediator. Consequently, to estimate the total effects of $E$ on $D$ in Fig. 1a, we would not control for the outcome risk factor ${R}_{0}$. If ${R}_{0}$ were to be controlled for, this would close the open path between $E$ and $D$ through ${R}_{0}$, leaving only the direct effect of $E$ on $D$.

When controlling for a mediator on the path between the exposure and outcome (for example by including it as a factor in a regression model), the resulting effect of the exposure on the outcome corresponds to what is known as a “controlled” direct effect [24]. At the population level, the controlled direct effect is defined as the average contrast between those with and without the exposure for a given value of the mediator [24]. Controlled direct effects can be estimated for both continuous and binary outcomes as well as for various effect measures, including odds ratio [25]. However, to obtain valid controlled direct effects for causal interpretation, at least two assumptions should be met [11, 12, 24–26]:

1. There should be no unmeasured exposure-outcome confounding. Although this type of confounding is common in observational research, it is uncommon in an immigrant dataset, given that few factors can influence which country one is born in.
2. There should be no unmeasured mediator-outcome confounding. This type of confounding is often ignored in the literature [27]. If unmeasured mediator-outcome confounding is present, adjusting for the mediator would lead to biased controlled direct effects due to conditioning on a collider.

In addition to these assumptions, any exposure-mediator interaction should be accounted for [11, 12, 24–26]. If an interaction is present but ignored in regression modeling, the estimated controlled direct effect would be biased. Further, if an exposure-mediator interaction is present, the controlled direct effect will vary by the levels of the mediator.

In this study, we do not discuss exposure-outcome confounding or exposure-mediator interaction further (details on these concepts can be found in Hernan et al. [20] and Rijnhart et al. [26], respectively). However, we will have a closer look at the implications of unmeasured mediator-outcome confounding (assumption 2.).

### Natural indirect and direct effects

For many research questions, the main goal is to decompose the total effect into direct and indirect effects using mediation analysis. That is, the goal is to assess the extent to which the effect of an exposure on the outcome is explained or is unexplained by a given set of hypothesized mediators. A common approach for this goal is to estimate the so-called natural direct and indirect effects [24]. This approach for estimating effect types differs from controlled direct effects in analysis techniques, conditions, and interpretations [11, 12, 24–28]. Specifically, the estimated natural direct and indirect effects are only valid when assumptions 1. and 2. above are met. In addition, there should be no unmeasured exposure-mediator confounding factors and no mediator-outcome confounding factors affected by the exposure. Note that, estimating natural direct effects will not be covered in this article as we are evaluating the implications of the common practice of regression adjustment in immigrant datasets, and not evaluating the various analysis techniques used for estimating direct or indirect effects. Details on natural direct and indirect effects can be found in T. J. VanderWeele [12].

All methods were performed according to relevant guidelines.

## Results

In the following, we investigate the underlying conditions leading to unequal distributions in the outcome risk factors between compared groups and show how the adjustment for outcome risk factors in regression models under these conditions can lead to either total effect or controlled direct effect.

### Model A: the outcome risk factor is both a selection factor and mediator

In Fig. 1b, we assumed that both the exposure and the outcome risk factor affected the probability of being a member of the immigrant dataset *S* (*E* → Ⓢ ← *R*_0_). This new structure added to Fig. 1b compared with Fig. 1a is an example of collider bias. In terms of DAGs, collider bias occurs when two variables point to the same variable (the collider), and this collider variable is adjusted for or conditioned on a specific value of its distribution [18, 21, 23]. In our DAG, $S$ is the collider and it is restricted to only those who are a member of the immigrant dataset ($S=1$), indicated by the circle around $S$. Importantly, conditioning on a single value of the collider $S$ opens the path which originally was closed (not shown in the DAGs), thus inducing a biased association between $E$ and ${R}_{0}$ [18, 21, 23].

However, in Fig. 1b, we also assumed that the countries of the immigrants and non-immigrants already predispose to a difference in the outcome risk factor ${R}_{0}$ before immigration took place, indicated by the path $E\to {R}_{0}$. Accordingly, the observed unequal distribution of the outcome risk factor between immigrants and non-immigrants consists of both a preexisting difference in the outcome risk factor as well as a biased association induced due to the selection process involving the outcome risk factor. This biasing part would further bias the effect of $E$ on $D$ due to the path *E* → Ⓢ ← *R*_0 _→ *R → D.* Consequently, to obtain valid effect estimates of the exposure on outcome, we need to remove the collider bias part of the observed unequal distribution between the compared groups.

In many situations, collider bias can be removed by simple regression modeling. To accomplish this, one needs to adjust for variables that lie on the biasing path between the exposure and outcome [18, 21, 29, 30]. In Fig. 1b, the outcome risk factor $R$ is the only measured variable that keeps the biasing path open between $E$ and $D$. Indeed, in the migration literature, it is common practice to adjust for $R$ in regression models due to the observed difference in $R$ between immigrants and non-immigrants. However, in doing this, we would not only remove the collider bias but also remove the indirect effect of $E$ on $D$ via $R$ ($E\to {R}_{0}\to R\to D$). Consequently, when the outcome risk factor is both a mediator and selection factor in immigrant datasets, the adjustment for $R$ would yield a controlled direct effect, and not a total effect, of $E$ on $D$. We later briefly discuss how total effects can be estimated under these conditions.

A challenge with adjusting for the outcome risk factor $R$ arises when there are also confounding factors $C$ for the relationship between ${R}_{0}/R$ and $D$ (Fig. 1c). Because ${R}_{0}$ is a close ancestor of $R$, and adjusting for $R$ therefore also largely adjusts for ${R}_{0}$, this adjustment may induce another collider bias due to the path $E\to {R}_{0}/R\leftarrow C\to D$. In this situation, additional adjustment for C is also needed to close this biasing path. If such mediator-outcome confounding is suspected but not measured and adjusted for, one should evaluate its potential impact on the estimated effects by sensitivity analyses [12].

### Model B: the outcome risk factor is a selection factor only

In Fig. 1a-c, we assumed that the countries of the immigrants and non-immigrants already predispose to a difference in the outcome risk factor ${R}_{0}$ before immigration took place. However, in some cases, this national ${R}_{0}$ distribution could be the same for the compared groups, with no arrow from $E$ to ${R}_{0}$ (Fig. 1d). This would for example be the case when ${R}_{0}$ represents the variable sex. In that case, the observed difference in the outcome risk factor distribution between groups is only attributed to collider bias due to the path *E* → Ⓢ ← *R*_0_. In other words, the outcome risk factor ${R}_{0}$ no longer acts as a mediator and remains a selection factor for immigration alone. Therefore, adjusting for the measured outcome risk factor $R$ using adjusted regression would appropriately close the collider path *E* → Ⓢ ← *R*_0_→ *R → D*, resulting in a total effect, and not a controlled direct effect, of $E$ on $D$.

Note that, in Fig. 1d the variable $C$ is a confounding factor for the association between ${R}_{0}/R$ and $D$. Once adjusting for the measured $R$, one may also need to adjust for $C$ to avoid another collider bias on the path ${R}_{0}\to R\leftarrow C\to D$. However, this bias may in general be small, as ${R}_{0}$ is a close ancestor of $R$, and adjusting for $R$ will largely also adjust for ${R}_{0}$ (and thereby close the confounding path ${R}_{0}\leftarrow C\to D$). Indeed, when $R$ is time constant (e.g., sex) or is measured at the point of immigration (setting ${R}_{0}=R$ in Fig. 1d), adjusting for $R$ would be sufficient to close all biasing paths including the path through the confounding factor $C$. In contrast, this would not suffice when $R$ is both a mediator and selection factor (setting ${R}_{0}=R$ in Fig. 1c), in which adjustment for $R$ would introduce collider bias due to the path $E\to R\leftarrow C\to D$. Then, additional adjustment for $C$ is needed to close the confounding path.

### Model C: the outcome risk factor is a mediator only

If the probability of being a member of the immigrant dataset does not depend on the outcome risk factor (i.e., no selection factor), we no longer have an arrow from ${R}_{0}$ to $S$ in Fig. 1b, c (not shown in the DAGs). In that case, the observed difference in outcome risk factor distribution between groups is only attributed to a pre-existing difference between countries before immigration took place. That is, the outcome risk factor now acts as a mediator alone for the association between country of birth and the health outcome ($E\to {R}_{0}\to R\to D$), and no adjustment for collider bias is needed. An example of such a mediator could be body height, which is unlikely to be a direct selection factor for being a member of an immigrant dataset. Consequently, adjusting for such an outcome risk factor would yield a controlled direct effect, like that seen in Fig. 1a. On the other hand, refraining from adjusting for the same outcome risk factor (when it is a mediator alone and not a selection factor) in regression models would lead to the estimation of total effect. Note that the controlled direct effect is only valid if also adjusting for potential mediator-outcome confounding factors.

### Model D: the outcome risk factor is neither a selection factor nor a mediator

If the outcome risk factor is neither a selection factor for immigration nor a mediator for the association of the exposure and outcome, the distribution of the outcome risk factor should be similar for the compared groups. In this case, adjustment for the outcome risk factor $R$ would not be needed to remove bias, and the effect estimates would be total.

### Note on post-migration change in outcome risk factors

If the outcome risk factors ${R}_{0}$ (before migration) and $R$ (some years post-migration) take different values, this could indicate that some factor associated with the receiving country may have caused this change. For instance, immigrants in the dataset can have their post-migration smoking status ($R$) changed compared to before immigration (${R}_{0}$), because the country where they move to have better education for citizens regarding the adverse effect of smoking, making these immigrants decide to stop smoking. Hence, there might be scenarios where $S$ can influence $R$, and where both $S$ and $R$ are mediators for the association between $E$ and $D$ due to the path $E\to S\to R\to D$. This new path will only have consequences for the estimated effects in Fig. 1d but not in Fig. 1b, c. In Fig. 1d, adjusting for $R$ and $C$ after inclusion of the arrow from $S$ to $R$ would lead to controlled direct effect rather than the previous total effect.

### Example of controlled direct effect

To illustrate how adjusting for outcome risk factors may lead to the estimation of a controlled direct effect instead of a total effect, we consider the study by Nilsen et al. [31] where the authors compared the risk of preterm preeclampsia (< 37 weeks of gestation) between immigrants and non-immigrants according to immigrants’ reasons for immigration to Norway. The study included seven immigrant groups, but here we compare only one of the immigrant groups (immigrant refugee women) with the non-immigrants.

The study reports results from two adjusted regression models. The first model adjusted for calendar year of birth, maternal age at birth, parity, marital status at birth, and chronic diseases (hypertension and diabetes). The second model additionally adjusted for SES, measured as maternal income and education. All variables were considered risk factors for preeclampsia and differed between the compared groups in initial data exploration in the immigrant dataset. Furthermore, they were measured post-migration around the time point of childbirth. For our illustration, we will assume that no other outcome risk factors existed for preeclampsia.

**Fig. 2:**
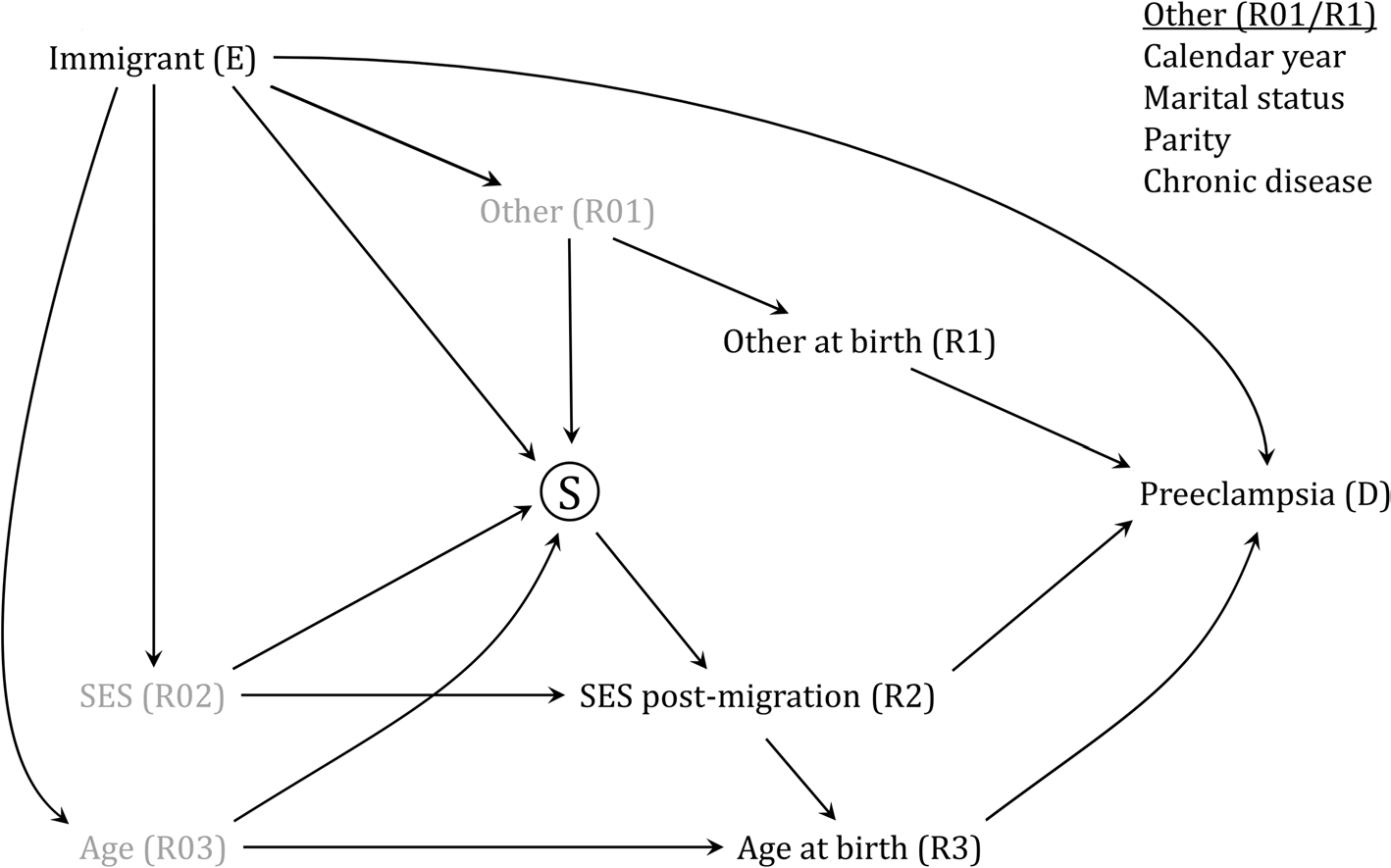
Example of controlled direct effect. Comparison of refugee immigrants and non-immigrants in terms of preeclampsia. See details in the text for interpretations. Abbreviations: SES, socioeconomic status

The suggested DAG for the fully adjusted model is shown in Fig. 2. The node $Immigrant\, (E)$ is the exposure and represents the groups being compared. The node Ⓢ represents the dataset containing both representative non-immigrant women and the subsample of selected refugees. Further, the gray nodes ($R01-R03$) represent unmeasured outcome risk factors pre-migration, while the black nodes ($R1-R3$) are measured risk factors post-migration. Also, we assume that the outcome risk factors $Calendar\, year$, $Marital\, status$, $Parity$, and $Chronic\, disease$, have the same causal roles, and have, therefore, combined these into $Other \left(R01\right)$ and $Other\, at\, birth\, (R1)$.

In essence, the DAG in Fig. 2 corresponds to that of Fig. 1b, where adjusting for all measured post-migration outcome risk factors ($R1-R3$) is needed to remove collider bias. However, in doing this, we also close all the indirect paths between exposure $E$ and outcome $D$ via these factors. Consequently, adjusting for the measured post-migration outcome risk factors should result in a controlled direct effect, and not a total effect, for the association between $Immigrant\, (E)$ and $Preeclampsia\, (D)$. Nilsen et al. [31] did not report the type of effect, and readers might therefore interpret the adjusted association as total effect rather than controlled direct effect.

It may be tempting to refrain from adjusting for the outcome risk factor when it is both a mediator and a selection factor, in the belief that the effect would be total. However, if the adjustment for such outcome risk factors is ignored, the estimated total effect will be biased due to uncontrolled collider bias. For example, the model with and without adjustment for SES in Nilsen et al. [31] produces two different odds ratios, 1.28 vs 1.18, for preeclampsia. The second estimate (with adjustment for SES) is a valid controlled direct effect, whereas the first estimate (without adjustment for SES) is a biased effect estimate due to ignoring SES as a selection factor.

It is likely that immigrants can have their post-migration $SES\, (R2)$ changed compared to before immigration ($R02$), indicated by the arrows from Ⓢ to $SES\, (R2)$ in Fig. 2. We further believe that SES post-migration may influence the age at which one decides to have a child, indicated by the arrow from $SES\, (R2)$ to $Maternal\, age\, (R3)$. However, because all measured outcome risk factors are mediators on the causal path from $E$ to $D$, and at the same time are adjusted for, these additional paths would not affect the effect type or results from the adjusted regression model.

## Discussion

**Fig. 3:**
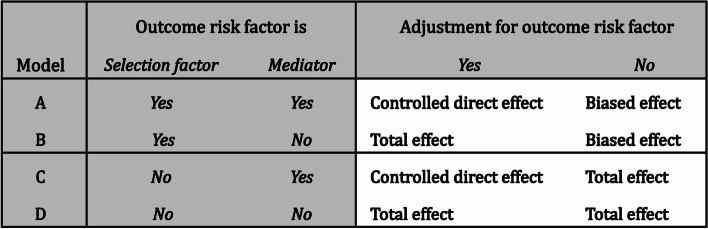
Total effect or controlled direct effect. Effect types obtained for the association between country of birth and health outcomes when adjusting for outcome risk factors that are either mediators, selection factors, or combined mediators/selection factors. See details in the text for interpretations

When quantifying difference in health outcomes between immigrants and non-immigrants, it is common practice to adjust for observed differences in outcome risk factors between the groups being compared. In this study, we showed that unequal distributions in the outcome risk factors between immigrants and non-immigrants arise due to various conditions involving the outcome risk factors. When the outcome risk factor acts as a combined mediator/selection factor or as a mediator alone, the regression adjustment for the risk factor leads to a controlled direct effect. When the outcome risk factor acts as a selection factor and not a mediator, the adjustment for the risk factor leads to a total effect. A summary of our findings is presented in Fig. 3. Notably, in most research problems, several different types of outcome risk factors can be present for the outcome. If at least one of these risk factors is a combined mediator/selection factor or a mediator alone, adjusting for the whole set of outcome risk factors would yield a controlled direct effect.

While the aim of this paper was to investigate how adjustment for outcome risk factors can lead to the estimation of either total or direct effects, we also described what happens if we do not adjust for selection factors or mediators. Specifically, we showed that refraining from adjusting for a combined mediator/selection factor or a selection factor alone can induce collider bias unless adjusting for some other measured factor on the same causal path (model A and B). Conversely, no such bias would be induced when refraining from adjusting for a mediator alone (model C) or a factor that is neither a mediator nor a selection factor (model D). The last column of Fig. 3 summarizes the implications of not adjusting for the relevant risk factor. In all models, also accounting for various confounding paths, including mediator-outcome confounding, may be necessary to obtain valid controlled direct effects or total effects.

If authors are not satisfied with controlled direct effects and want to obtain total effects under model A (the outcome risk factor is both a selection factor and mediator), other statistical techniques could be used for this purpose. One popular analytical approach would be to use inverse probability weighting (IPW) in regression models [22, 29, 30]. To use this method, we first need to calculate the probability ($p$) of being a member of the immigrant dataset ($S = 1$) for each individual and $R$ value. Then, we calculate the inverse of these selection probabilities ($1/p$) and use these as weights in regression models. Note that, however, to calculate the selection probabilities for IPW, we need data on both the outcome risk factor distribution for the immigrants in the sample of the receiving country (immigrant dataset) as well as the corresponding distribution in the home population of the immigrants (i.e., the national representative data). This requirement is, unfortunately, not always possible to meet.

In our analyses, we considered national representative data for the non-immigrants. However, many researchers may not have access to nationally representative data of non-immigrants but are left with a survey in which both the immigrants and non-immigrants select themselves to be members of the immigrant dataset ($S = 1$). This would not change the DAG in Fig. 1b-d, and the approach for estimating total effects and controlled direct effects would be the same as before.

## Conclusions

In immigrant datasets, adjusting for outcome risk factors in regression models may result in either total effects or controlled direct effects. Which type of effect is estimated under a given dataset depends on the causal role of the outcome risk factor adjusted for. Because total and direct effects are two different effects and are interpreted differently, we advise researchers to clarify to the readers which types of effects are presented when adjusting for outcome risk factors in immigrant datasets. As shown in this study, this can best be accomplished by first examining the plausible model for the research problem using causal graphs and then identifying the correct effect type obtained by adjustments under these models. Only this way, the readers may achieve a consistent interpretation of effects and perform consistent comparisons between immigrants and non-immigrants across immigrant datasets. The current paper is focused on immigrant datasets, but the content of the paper may also be relevant to other public health datasets, including datasets of health difference between males and females.

## Data Availability

Not applicable.

## Abbreviations

BMI: Body mass index
DAG: Directed acyclic graphs
IPW: Inverse probability weighting
SES: Socioeconomic status
